# Optimization of breast cancer excision by intraoperative ultrasound and marking needle - technique description and feasibility

**DOI:** 10.1186/s12957-015-0568-8

**Published:** 2015-04-18

**Authors:** Nebojsa S Ivanovic, Darko D Zdravkovic, Zlatko Skuric, Jelena Kostic, Natasa Colakovic, Miodrag Stojiljkovic, Svetlana Opric, Magdalena Stefanovic Radovic, Ivan Soldatovic, Biljana Sredic, Miroslav Granic

**Affiliations:** Department of Surgical Oncology, UMC Bezanijska Kosa, Autoput bb, Belgrade, 11000 Serbia; Medical Faculty of Belgrade University, Dr Subotica 8, Belgrade, 11000 Serbia; Department of Pathology, UMC Bezanijska kosa, Autoput bb, Belgrade 11000 Serbia; Department of Radiology, UMC Bezanijska kosa, Autoput bb, Belgrade, 11000 Serbia; Department of Statistics, Medical faculty of Belgrade University, Dr Subotica 8, Belgrade, 11000 Serbia

**Keywords:** Intraoperative ultrasound, Marking needle, Breast-conserving surgery, Breast cancer

## Abstract

**Background:**

We present a surgical technique and the preliminary results of breast cancer excision after insertion of a specially constructed marking needle into the tumor, controlled by intraoperative ultrasound.

Resection margins were projected in six directions by ultrasound measurements, determined in relation to the needle, and resection was done in accordance with those measurements. The main objective was to obtain resection margins similar (equal) to those projected by intraoperative ultrasound (10 mm).

**Methods:**

Detailed description of the technique is given. Thirty-two female patients undergoing breast-conserving surgery, up to 30 mm in diameter, for palpable and non-palpable invasive breast cancer, were operated on using this technique. Its feasibility was tested by analyzing the success (rate) of needle placement in the tumor, the measurements executed, and the performance of the excision.

**Results:**

All stages of the technique were successfully performed to completion on all 32 patients. The procedure of needle placement and ultrasound measurement of distances took 11 min on average (between 6 and 20 min). The average distance of the tumor margin from the resection margin was 12.9 mm (2 to 30 mm, 95% confidence interval [11.9, 14.06]). There was one patient with a positive resection margin (3%).

**Conclusions:**

The technique of excising palpable and non-palpable breast cancer by intraoperative ultrasound and an especially constructed marking needle is feasible and comfortable to perform. Preliminary results imply that resection volume can be rationalized, with the same or better oncological safety.

## Background

Intraoperative ultrasound (IOUS) in breast tumor surgery was introduced into clinical practice as a means of localizing non-palpable tumors [1-12] and more recently has been used in the surgery of palpable tumors [13-21], with the aim of optimizing resection procedures and overcoming the shortcomings of classic palpation-guided surgery. In descriptions of the technique of IOUS application, most authors rely on direct contact between the ultrasound probe and the tumor specimen. Using the IOUS in this, let it be called, standard way, produces some difficulties that disturb the comfort and accuracy of both procedure and performance: the presence of air and liquid in the wound may create content which significantly disturb the quality of the ultrasound image; insertion of a probe into the wound can mean movement and pressure of the tissue, which may in turn influence the assessment of resection margins; and refraction of ultrasound waves while passing through tissue of irregular shape, such as a tumor, may create a difference between the size of tumor measured by ultrasound and the real size [22].

These difficulties motivated us to create a technique of resection for both palpable and non-palpable breast tumors by using a marking needle which is inserted into the tumor and is guided by ultrasound during the operation, while the patient is anesthetized. Measurements of the distance of resection margins are performed using ultrasound in relation to this needle, in all directions. The resection is thus performed according to these measurements, with continual measurement of the resection margins in relation to the needle using a sterile ruler.

## Methods

By the end of October 2013, 32 patients with palpable and non-palpable unifocal breast carcinoma (29 palpable and 3 non-palpable) with diameters of up to 30 mm, visible by ultrasound, had been operated on, using the presented technique, in the Department of Surgical Oncology at UMC Bezanijska Kosa. All operations were performed by one surgeon, using a 7.5 MHz US probe, Hitachi EUB 5500 (Tokyo, Japan). The diagnosis of breast carcinoma was made by a frozen section technique in all cases, after tumor excision. The time was recorded from the beginning of ultrasound measurement to the moment of incision. All patients have signed the informed consent.

The aim was to test the feasibility of the technique. The main parameters of success were:

1. Successful needle insertion into the tumor (that is, the presence of the needle in the tumor at least 2 mm from the closest margin).
2. Successful performance of the resection procedure (the needle at completion of resection should be in the same position as it was at the beginning, checked by ultrasound examination of the specimen at the end of excision).
3. Good correlation between distances from the tumor border to the resection margin projected by ultrasound (always 10 mm in six directions), measured on a histological specimen by a pathologist.
4. An acceptable percentage of positive resection margins (microscopic presence of tumor cells at 2 mm and less from the resection margin).

### Technique description

**Figure 1:**
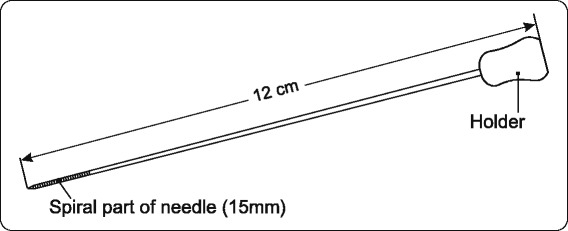
Marking needle.

The operation starts by marking the point of tumor projection on the skin by US-imaging. The skin incision is planned according to that point, applying standard directions of incision in breast cancer surgery. After the incision line is drawn, a probe is positioned with its longer axis on that line. To visualize the tumor in its largest diameter, the US probe is moved in soft transversal motions, perpendicular to the long axis of the transducer. The tumor diameter (transverse versus vertical), the lesion-to-skin distance, and the lesion-to-fascia distance are measured in millimeters. This process is repeated in the cranial-caudal plane. The probe is again positioned over the incision line and kept there in order to monitor the insertion of the needle into the tumor (Figure 1).

### Needle insertion

The skin puncture point for inserting the needle is the starting or finishing point of incision. We always puncture the tumor in a laterally medial or mediolateral direction.

**Figure 2:**
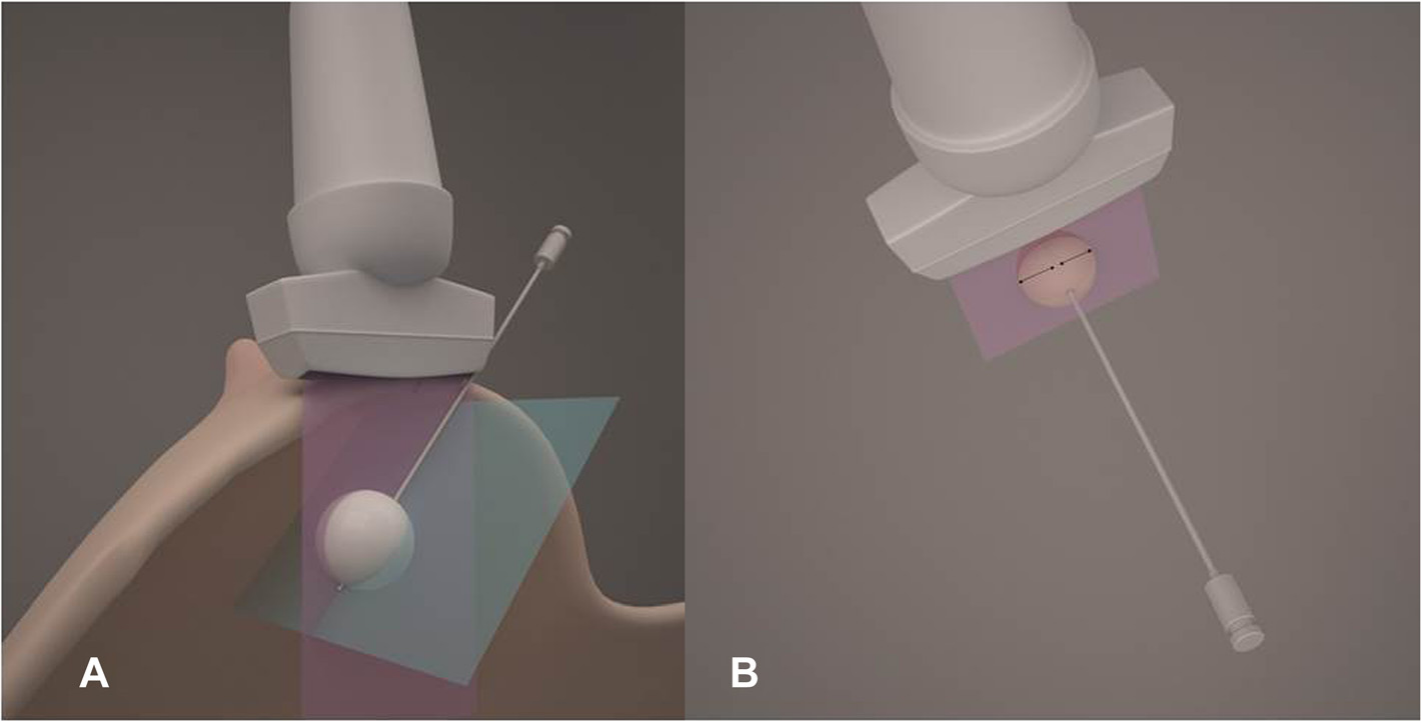
The needle in the center of the tumor in two orthogonal planes **(A)** and probe orthogonally to the needle axis **(B)**.

After making a 2- to 3-mm incision, the needle passes through the skin and punctures the breast tissue on the plane of the ultrasound wave, taking the direction of the central axis of the tumor (Figure 2A).

The needle is inserted into the central axis of the tumor (or as close as possible to the central axis) as seen from the skin toward the pectoral fascia.

The central position of the needle in the plane orthogonal to this plane was achieved earlier, by positioning the needle in the plane of the ultrasound wave. The needle passes through the whole length of the tumor. Its tip should be at the distal tumor margin, or a few millimeters in front of, or beyond the distal margin. When the needle is localized in the desired position, necessary measurements are performed (Table 1, Figure 2B).

**Table 1:** **Measurements performed during surgery**

**Measurements**	**Areas**
Tumor diameters	Skin-fascia
	Mediolateral
	Cranial-caudal
Tumor distances	Surface margin-skin
	Deep margin-fascia
Depth of the needle insertion (distance from the point of the needle to distal tumor margin)	The point inside the tumor
	The point beyond the distal margin
Distance from the needle to tumor borders	Needle-surface border (toward skin)
	Needle-deep border (toward fascia)
	Needle-cranial border
	Needle-caudal border

**Figure 3:**
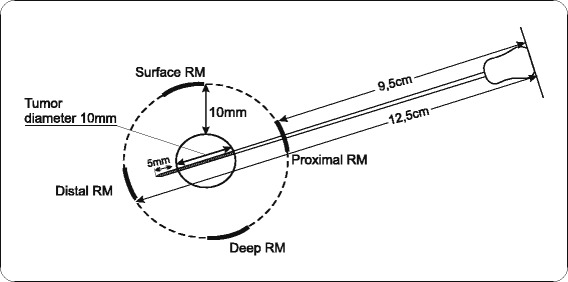
The point of needle reaches over tumors distal border by 5 mm.

By adding of 10 mm to each of these values, all resection margins are precisely determined, that is, their distance from the needle is established (Figure 3).

### Resection procedure

Incision of the skin is made along the previously planned line. All measurements (distance from the needle to resection margins) are performed using a sterile ruler.

The breast tissue is cut along the incision line until the needle fixed in the tissue is reached, proximal from the tumor. The distance of this point from the starting point of needle is determined. Then, by incising the tissue along the length of the needle, we reach the specimen at the point of the proximal resection margin along the length. The procedure is then continued by incising the breast tissue along the whole length of the incision, to the distal resection margin, and to a depth representing the surface resection margin, at an appropriate distance, from the needle toward the skin.

**Figure 4:**
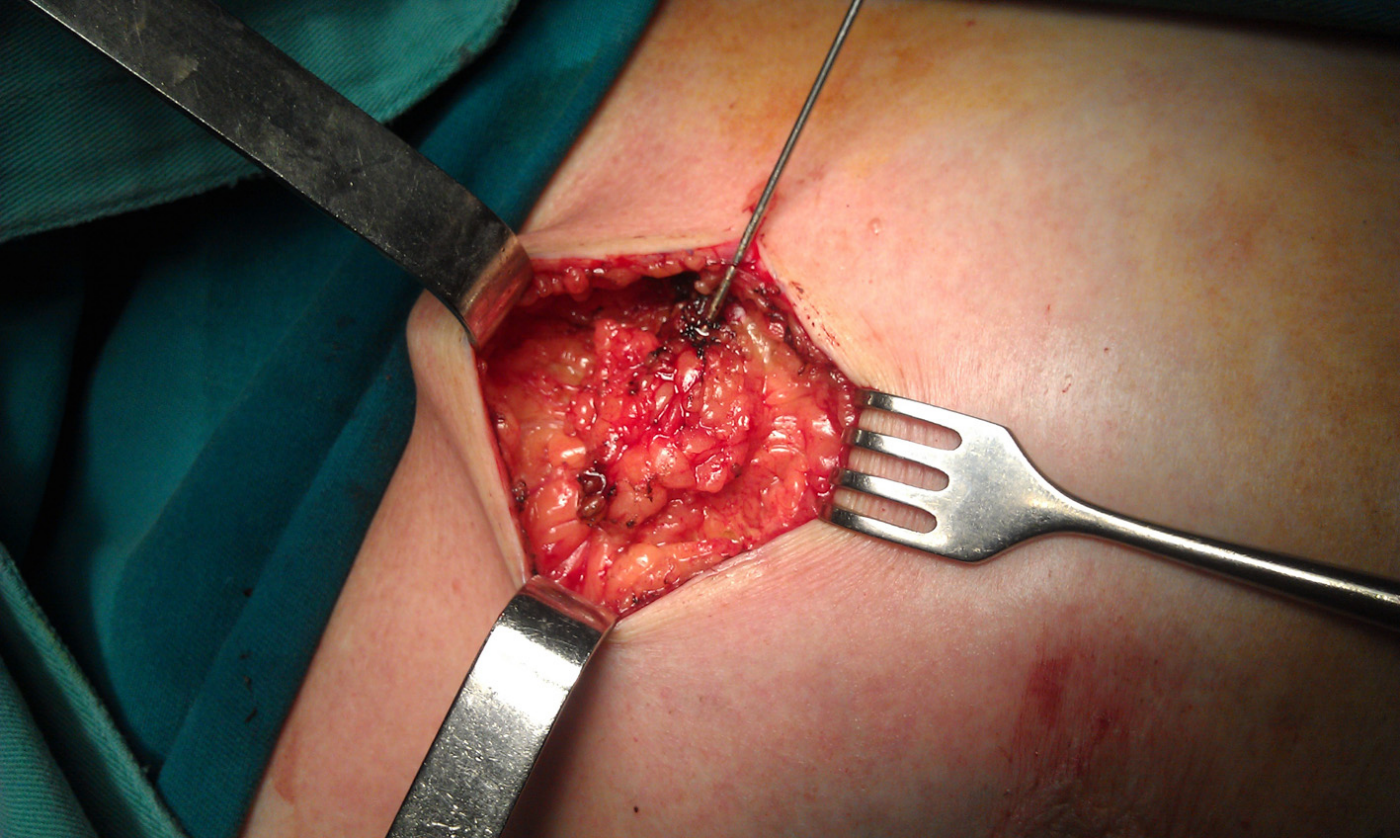
Resection procedure.

**Figure 5:**
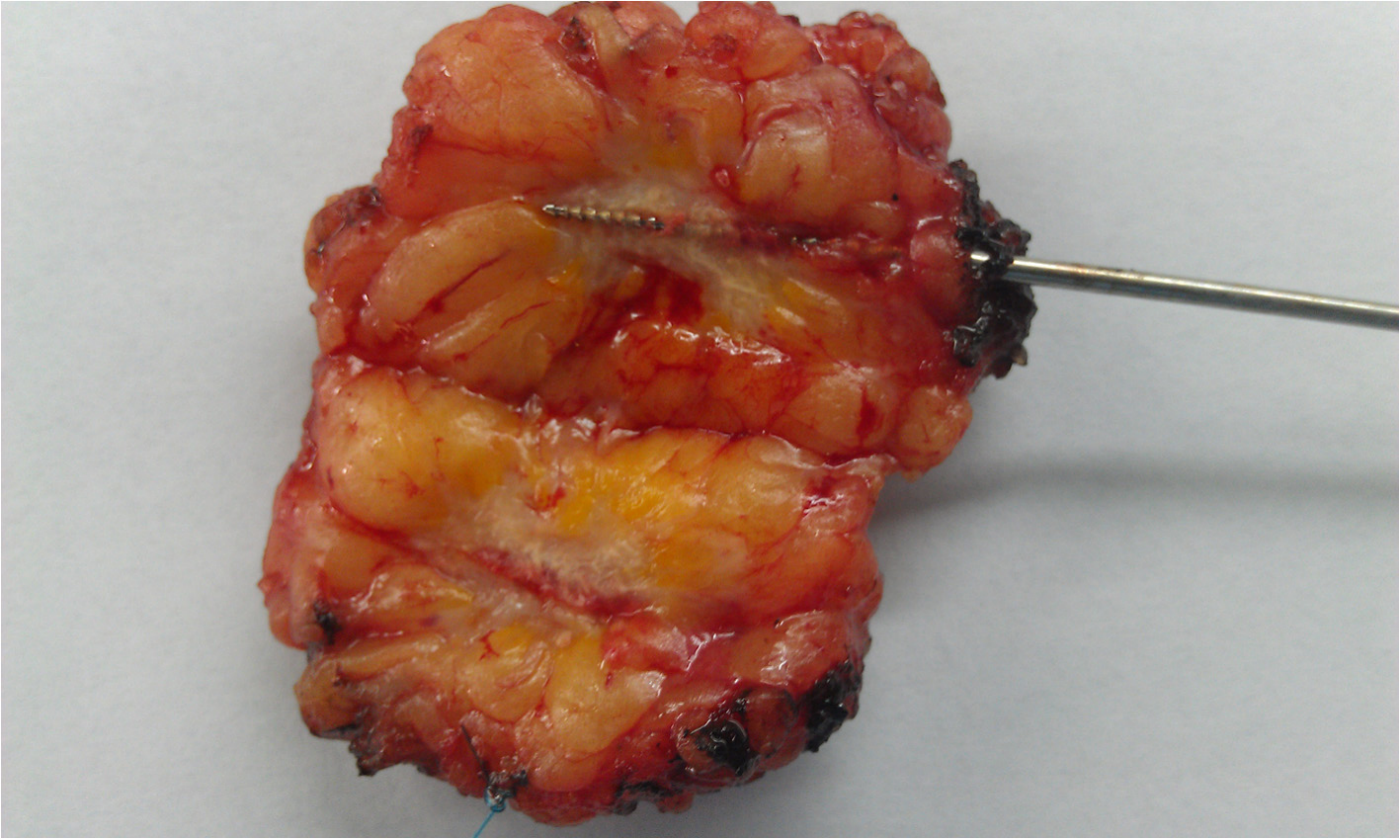
Specimen and tumor cut along the needle, which is still in the tumor.

From this line, resection extends laterally (cranially and caudally) in both directions from the needle until an appropriate distance to resection margins is reached (Figure 4). This continues to the deepest resection margin (toward the pectoral fascia). When resection is completed, the specimen is released from the surrounding tissue and is positioned on the needle, as on a spit (Figure 5). The specimen is then spatially oriented and sent to histopathology for processing and analysis (Table 2).

**Table 2:** **Measurements performed during histopathology processing**

**Measurements**	**Areas**
Tumor diameters	Skin-fascia
	Mediolateral
	Cranial-caudal
Specimen diameters	Skin-fascia
	Mediolateral
	Cranial-caudal
Resection margins	Toward skin
	Toward fascia
	Medially
	Laterally
	Cranially
	Caudally

## Results

A total of 32 female patients were included in this study (Table 3).

**Table 3:** **Patient and tumor characteristics**

	**Number of patients**	**%**
Tumor localisation		
Upper outer quadrant	14 (1 non-palpable)	43.75
Upper inner quadrant	6	18.75
Lower outer quadrant	8 (2 non-palpable)	25.0
Lower inner quadrant	4	12.5
Tumor type		
Invasive ductal carcinoma	28 (3 non-palpable)	87.5
Invasive lobular carcinoma	4	12.5
Axillary surgery		
Sentinel lymph node biopsy	25 (3 non-palpable)	78.0
Axillary lymph node dissection	7	22.0

Mean tumor volume (three diameters multiplied) in cm^3^ (range): 4.4 (0.28 to 15.12); (volume of non-palpable tumors: 0.28, 0.336, and 0.448 cm^3^).

In all cases, the tumor was successfully punctured (100%) and resected (the needle being in the same position inside the tumor at the end of procedure as it had been at the beginning (100%)).

The average time from the beginning of measuring to incision was 11 min (6 to 20 min).

The average distance of the tumor margin from the resection margin was 12.9 mm (95% confidence interval, 11.9 to 14.06). In six cases, when resection margins were 5 mm and less (3 times at 5 mm, twice at 4 mm, once at 3 mm, and once at 2 mm), additional shaving excisions were performed.

There was one patient with a positive resection margin (3%).

## Discussion

The technique described here represents an effort to make resection procedures in breast-conserving surgery (both for palpable and non-palpable tumors) more objective and measurable, with the objective of achieving full tumor removal with the smallest possible loss of sacrificed healthy tissue around the tumor.

It is clear that surgery done by palpation cannot improve the objectivity and measurability of resection procedures. If we want to improve objectification and optimisation of excision in breast cancer, we have to make both the tumor and the surrounding tissue visible and measurable in real time, during the operation itself. The only means available to meet this demand is intraoperative ultrasound.

Studies published thus far have shown extraordinary success with intraoperative ultrasound in localizing non-palpable tumors, as well as advantages in relation to widely accepted WNL [1-12,20,23-26]. More recently, ultrasound measuring of resection margins has proved useful and efficient in planning and performing resections of palpable tumors [14,17,19]. Previously described techniques of using ultrasound in planning resection margins in palpable tumors rely on direct contact between the ultrasound probe and the specimen, without a marker visible by ultrasound. In our experience, such an approach is burdened with difficulties, especially inaccuracy in visualization due to non-homogeneous content in the wound and tissue movement during surgical work.

The technique that we propose means introducing a specially designed marking needle into the tumor, visible by ultrasound, in relation to which distances of tumor margins are measured in all directions, as well as the desired resection margins. The needle thus placed represents guaranteed unchangeability of dimensions, and visualization is ideal before the incision is made.

A preoperative core biopsy should be avoided because the resulting hematoma could affect the US visualization, and tumor damage could affect stability and firmness when anchoring the marking needle. We therefore did the frozen section procedure in all cases.

Our preliminary results show that the technique is feasible. In all cases, tumors were successfully punctured, and all necessary measurements, as well as the resection itself, were performed successfully. The only shortcoming of this procedure may be the additional time needed for measuring before incision (about 11 min on average).

## Conclusions

Intraoperative US with a marking needle allows real-time localization of breast carcinoma and subsequent planning of surgical margins, thereby resulting in objective, measurable tumor excision. In a randomized clinical trial, intraoperative US with marking needle guidance for palpable and non-palpable breast cancer will be evaluated for margin clearance, excision volume, cosmetic outcomes, and quality of life. The results will be compared with palpation-guided surgery.

## Abbreviations

IOUS: intraoperative ultrasound
RM: resection margin
UMC: university medical center
US: ultrasound
WNL: wire needle localization.
